# Childhood trauma, psychache, and depression among university students: a moderated mediation model

**DOI:** 10.3389/fpsyt.2024.1414105

**Published:** 2024-06-24

**Authors:** Shufeng Chen, Tiantian Fu, Yiwen Wang, Guoxiao Sun

**Affiliations:** School of Physical Education, Shandong University, Jinan, China

**Keywords:** childhood trauma, ambivalence over emotional expression, physical activity, depression, psychache

## Abstract

**Background:**

Childhood trauma is a potential threat to depression and can have a lifelong impact on the mental health of university students. Our study aimed to construct a moderated mediation model to explore the relationship between childhood trauma, psychache, ambivalence over emotional expression, physical activity, and depression in university students.

**Methods:**

A cross-sectional study was conducted in three universities in China, recruiting 476 university students using self-report questionnaires. The moderated mediation model was examined using the SPSS PROCESS model 21.

**Results:**

Ambivalence over emotional expression (F=12.843), childhood trauma (F=117.639), and psychache (F=581.594) all had a significant positive effect on depression (p<0.001), explaining 2.9%, 21.7%, and 56.8% of the variance, respectively. On the chain of influence between childhood trauma and depression, the mediating effect of psychache, the moderating effect of ambivalence over emotional expression, and the moderating effect of physical activity are all significant the overall indirect effect value of the three is 0.287, accounting for 61.59% of the total effect.

**Conclusion:**

This study investigated the relationship between childhood trauma, ambivalence over emotional expression, psychache, physical activity, and depression in university students. Future interventions should focus on developing good emotional expression among university students, increasing opportunities for physical activity, and reducing psychache to reduce depression.

## Introduction

Depression is characterized by slowed thinking, diminished interest, reduced verbal activity, low mood, loss of pleasure, suicidal ideation, and various other symptoms (1, 2). A significant amount of evidence has shown that depression severely threatens people’s mental and physical well-being worldwide (3). Notably, there has been a recent surge in unfavorable mental health conditions (4). According to the World Health Organization (WHO), depression will emerge as a significant health concern, a primary determinant of severe illness, and mortality by 2030 (5, 6). Studies have shown that the mean age of university students falls in a period of vulnerability for development of mental health problems. During this stage, students are transitioning from adolescence to adulthood, needing to adapt to new living environments and face academic pressure and competition. Due to these multifaceted pressures, they often encounter difficulties and challenges, making them more prone to mental health problems. Therefore, the university students may represent a representative sample in mental health research (7). It is estimated that 70% of people with depression have experienced at least one form of childhood abuse, and more than one-third of the world’s population has experienced adverse childhood experiences (8). Therefore, a more in-depth study of the factors influencing depression among university students is of great significance for personal growth and the development of the family and society.

Childhood trauma (CT) refers to various traumatic events individuals suffer during childhood and adolescence, including psychological, physical, or sexual abuse, as well as neglect and bullying (9). Adverse childhood experiences are strongly associated with individuals suffering from depression (10). Attachment theory points out that individuals tend to focus on critical others (e.g., parents) for closeness and protection. Individuals who have experienced childhood emotional neglect, whose internal working model significantly impacts the individual’s cognitive, emotional, and behavioral adjustment, are more likely to develop an unstable attachment style (11). Jaye et al. (12) found that childhood abuse positively associated with university students’ depression, suicidal ideation, and suicide attempts. The longer the abuse lasted, the higher the level of depression in adulthood would be. Based on previous attachment theories, we propose hypothesis 1: Within the university student population, greater levels of childhood trauma are associated with increased severity of depression.

Psychache is an experience of mental distress, such as guilt and despair, caused by a blocked psychological need (13). According to Shneidman’s Psychache theory, psychache is a distinct type of pain, different from normal physical pain, caused by psychological and emotional trauma that cannot be medically proven. Adverse life events are associated with an individual’s level of psychache, which can be significantly increased when an adverse life event is experienced, eventually leading to suicide (13). Psychoanalytic theory points out that trauma experienced in childhood can leave a mark on the psyche. If not dealt with appropriately, these marks will appear differently as individuals grow up, eventually developing severe mental health problems (14). Parental abuse of children can cause both internalizing and externalizing psychological problems, and the severity of the psychological issues increases with the extent of abuse and neglect (15). Adolescents troubled by CT can develop negative psychological perceptions and feelings of tiredness, leading to psychache (16). Negative Emotion Avoidance theory states that individuals who experience CT make negative attributions of failures and circumstances (17). The more CT a person experiences, the more psychache they feel, and the more likely they are to develop depression (18). Some studies have found that psychache can mediate the relationship between many variables and depression (19), with data suggesting that a lack of ability to manage psychache can lead to depression and ultimately to suicide (20). The Hopelessness Theory of Depression posits that certain individuals possess a cognitive vulnerability to depression, heightening the risk of depressive episodes following negative life events (21). These negative cognitive tendencies predispose individuals to feelings of despair, which the theory suggests is the primary catalyst for depression. Drawing from this theory, we propose hypothesis 2: Higher levels of psychache in university students correlate with increased severity of depression. Furthermore, hypothesis 3: Childhood trauma influences depression through the mediating role of psychache.

Ambivalence over emotional expression (AEE) is a personality trait or disposition. There is an ambivalence between the desire to express and the inability to express one’s genuine emotions. Emmons and Colby (22) suggested that individuals with high AEE have more negative self-perceptions and more subjective perceptions of their experiences as unfavorable and therefore experience more psychache. Individuals who are high in AEE are less likely to perceive empathy and help from others, and they are more likely to experience psychache as a result of experiencing CT (23). When external stimuli reach their threshold level, they develop a corresponding cognitive regulation pattern (24, 25). Individuals with AEE desire to express genuine emotions but fear that expressing true emotions will lead to negative consequences. Thus, they may adopt negative cognitive strategies to resolve internal conflicts (26). Porter (27) has found that patients with high AEE experienced more painful catastrophes, reporting increased pain and happiness. The higher AEE, the more pronounced the negative emotions and psychache (28). Personality Trait theory points out that individuals with different personality traits experience emotions differently when faced with external information or stimuli due to differences in levels of neurological arousal (29). Thus, individuals with high AEE may have lower levels of arousal of the corresponding neural mechanisms, thus be more sensitive to negative stimuli (30), and have greater levels of psychache (26). Based on Personality Trait theory, we propose the hypothesis 4a: AEE moderates the positive relationship between CT and psychache.

Based on a perspective of Emotion Regulation theory, Gross (31) pointed out that individuals regulate their emotions by integrating multiple factors interconnected with coping and self-esteem. Exercise is a holistic coping regulation that can regulate emotions and promote psychological well-being (32). Research in exercise psychology has shown that physical activity (PA) enhances physical fitness and improves mood, with higher levels of fitness gaining faster recovery rates in the cardiovascular system in response to psychache (33). The cognitive view of physical activity on mood regulation mechanisms points out that PA can improve mental health by triggering positive emotions and resisting negative states of mind, such as depression, anxiety, and confusion (34). PA of university students is strongly associated with negative emotions, and low PA is linked with a higher risk of depression compared to high PA (35). Therefore, this study proposes hypothesis 4b: PA moderates the positive relationship between psychache and depression.

**Figure 1:**
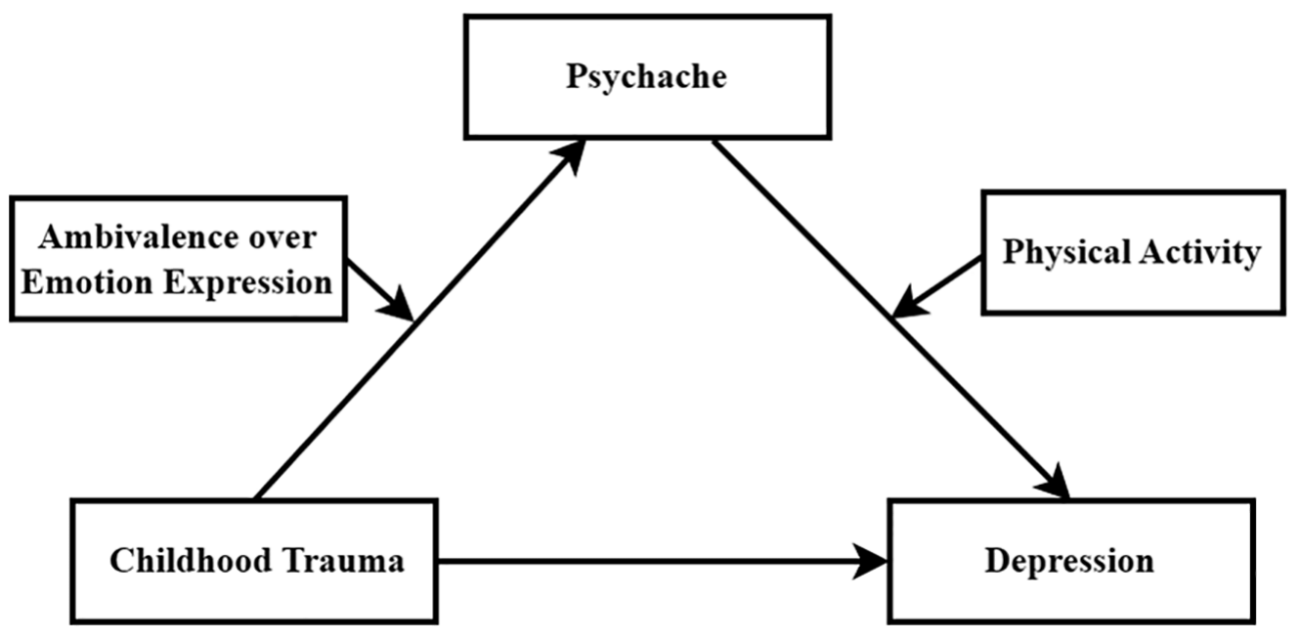
Hypothetical models.

This study aims to investigate the factors that influence the association between CT and depression, which will help to develop effective interventions for depression. To achieve the study’s objectives, a moderated mediation model was constructed and is presented in **Figure 1**.

## Methods

### Participants and procedures

Participants in this study were university students. Data were collected from Shandong, Liaoning, and Guizhou between October and December 2022. The questionnaires were distributed through the Questionnaire Star platform (http://www.wjx.cn). All participants gave informed consent, and the purpose and methods of the study were explained in detail before participation. Quality control was performed during the survey, and questionnaires with invalid, incomplete, or contradictory responses were excluded after the survey was completed. Data from 476 participants were finally included in the analysis. The studies involving human participants were reviewed and approved by the School of Public Health Ethics Committee at Cheeloo College of Medicine of Shandong University (20190912). The mean age of the participants was 20.32 years (*SD*=2.327, range 17 to 22 years). The specific data is presented in **Table 1**.

**Table 1:** Demographic variables (*N*=476).

Variables	Category	*N*	*%*
Gender	Male	284	59.7
	Female	192	40.3
Residence	Rural	267	56.1
	Urban	209	43.9
University course	Liberal arts	192	40.3
	Sciences	284	59.7
Grade	Freshman	144	30.3
	Sophomore	110	23.1
	Junior	75	15.8
	Senior	76	15.9
	Postgraduate	71	14.9
Only child	Yes	193	40.5
	No	283	59.5

### Measures

#### Demographic variable

Age, gender, university course, grade, place of residence and being only child or not were collected as sociodemographic variables.

#### Childhood trauma

The Short Form of the Childhood Trauma Questionnaire (CTQ-SF) was developed by Bernstein et al. (36). A 5-point Likert-type scale is used, with scores ranging from 1 (“never”) to 5 (“almost always”). The higher the total score, the more severe the trauma. The questionnaire is reliable in our sample, with a Cronbach’s alpha coefficient of 0.862 in this study.

#### Ambivalence over emotion expression

The Emotion Expression Conflict Questionnaire (AEQ) was developed by King and Emmons (26) and modified by Feng et al. (37). The scale has 23 entries and includes five dimensions: inhibition of positive emotional expression, inhibition of negative emotional expression, emotional myths, desire to be understood, and expression of regret. The questionnaire employs a 7-point Likert-type scale ranging from 1 (“totally disagree”) to 7 (“absolutely agree”), with higher scores indicating higher degrees of AEE for the individual. Cronbach’s alpha coefficient for this scale in this study was 0.924.

#### Physical activity

The Physical Activity Rating Scale (PARS-3) was revised by Liang (38). The scale consists of three hierarchical questions investigating three dimensions of PA: intensity, duration, and frequency. The higher the total score, the higher the standard of PA. The Cronbach’s alpha coefficient of 0.761 in this study.

#### Psychache

The Psychache Scale (PAS) was developed by Holden et al. (39) to measure the level of psychache in the university population. The 13-item scale uses a 5-point Likert-type scale, with scores ranging from 1–5, indicating “never” to “almost always”, with higher scores indicating higher psychache levels. The questionnaire has good applicability among university students in China (40), and Cronbach’s alpha coefficient of 0.916 in this study.

#### Depression

The Center for Epidemiologic Studies Depression Scale was developed by Radloff (41). The purpose is to measure the degree of current depression symptoms, including four dimensions of depression, positive mood, somatic symptoms, activity retardation, and interpersonal relationships. The questionnaire consists of 20 items, each rated on a 0 (occasionally or not) - 3 (most of the time), a 4-point Likert-type scale. The total score is 0–60, with higher scores indicating a greater likelihood of depression symptoms. The Cronbach’s alpha coefficient for this scale in this study was 0.906, with good reliability.

### Statistical analysis

The data analysis was conducted using SPSS 24.0, and the preprocessing steps included centering the data and reversing the scored items. Key variables’ distributions were shown using descriptive statistics, including frequencies, means, and standard deviations, and the relationships between variables were examined using Pearson correlation analysis. Analyses were conducted using Model 4 of SPSS PROCESS 4.1 (http://www.afhayes.com) developed by Hayes (42) to test the mediating effect of CT on depression under the influence of psychache, and PROCESS (Model 21) to assess the mediating role of psychache, the moderating effect of AEE on childhood trauma and psychache, and the moderating effect between PA and psychache. A moderated mediation model was constructed to test the hypotheses of this study. The indirect effect of childhood trauma on depression was analyzed using the Bootstrap method, with a sample of 5000 drawn from PROCESS. The effect’s 95% confidence intervals (*CIs*) were determined, with significant effects indicated if the confidence interval did not include 0.

## Results

### Demographic variables

This study collected sociodemographic variables including age, gender, university course, grade, place of residence and only child status. The mean age of the participants was 20.32 years (SD=2.327, range 17 to 22 years). Among the participants, 284 (59.7%) were male and 192 (40.3%) were female; 192 (40.3%) were in liberal arts, and 284 (59.7%) in sciences; 267 (56.1%) lived in rural areas and 209 (43.9%) in urban areas. Additionally, 193 (40.5%) were only child, and 283 (59.5%) were not; 144 (30.3%) were freshmen, 110 (23.1%) were sophomores, 75 (15.8%) were juniors, 76 (15.9%) were seniors, and 71 (14.9%) were postgraduates. The specific data is presented in **Table 1**.

### Common method bias analysis

This study conducted a factor analysis on all questionnaire items using Harman’s one-way test to test for potential standard method bias (43). Bartlett’s test of sphericity was significant (KMO = 0.953, p<0.001) through principal component analysis, with a total of 12 factors having eigenvalues greater than 1. The variance explained for the first factor was 29.44% (below the critical indicator of 40%), demonstrating that the questionnaire has no widespread methodological bias (44).

### Direct effects of CT, AEE, psychache, and PA on depression

We finally analyzed data from 476 participants. The mean, standard deviation, and correlation analyses for each variable are shown below in **Table 2**. CT was significantly positively correlated with psychache (*r*=0.393, *p*<0.01) and depression (*r*=0.466, *p*<0.01), AEE was significantly and positively correlated with psychache (*r*=0.158, *p*<0.01) and depression (*r*=0.171, *p*<0.01), psychache was significantly and positively correlated with depression (r=0.760, p<0.01), PA was not significantly with psychache (*r*=-0.002, *p*>0.05) and depression (*r*=-0.058, *p*>0.05).

**Table 2:** Correlations between the main study variables (*N*=476).

variables	*M*	*SD*	CT	AEE	Psychache	Depression	PA
CT	38.24	14.056	1	–	–	–	–
AEE	88.55	36.33	-0.036	1	–	–	–
Psychache	22.45	11.845	0.393^**^	0.158^**^	1	–	–
Depression	16.73	10.549	0.466^**^	0.171^**^	0.760^**^	1	–
PA	28.37	25.874	-0.033	-0.071	-0.002	-0.058	1

CT, childhood trauma; AEE, ambivalence over emotion expression; PA, physical activity. **p < 0.01

Several regression equations were established using forced entry method with depression as the dependent variable and AEE, PA, CT, psychache, and the interaction effect between psychache and PA as the independent variables. The results, as shown in **Table 3**, indicated that AEE (F=12.843), CT (F=117.639), and psychache (F=581.594) had a significant positive effect on depression (p<0.001), accounting for 2.9%, 21.7%, and 56.8% of the variance, respectively. The interaction effect between psychache and PA (F=132.189) also had a significant positive impact on depression (p<0.001), accounting for 23.8% of the variance. However, PA (F=1.434) did not have a significant effect on depression (p>0.05).

**Table 3:** Regression analysis of CT, Psychache, AEE, and PA on depression.

Variables	Depression					
	B	SE	β	T	F	R^2^
CT	0.350	0.032	0.466	10.846	117.639^***^	0.217
Psychache	0.677	0.028	0.760	24.116	581.594^***^	0.578
AEE	0.050	0.014	0.171	3.584	12.843^***^	0.029
PA	-0.704	0.588	-0.058	-1.197	1.434	0.003
Psychache×PA	0.163	0.014	0.488	11.497	132.189^***^	0.238

CT, childhood trauma; AEE, ambivalence over emotion expression; PA, physical activity. ^***^p < 0.001

### Indirect effects of CT, AEE, psychache, and PA on depression

As this model is a mixed model with both mediating and moderating variables, ordinary regression analysis cannot accurately reveal the mechanisms of influence among the variables. Therefore, a more in-depth mediation effect test is required. This study used Bootstrap mediation test and 95% confidence intervals, based on 5000 Bootstrap samples to test the mediating effect. Since the control variables have no significant effect on the model, they are not shown in the table, and the main focus of the table is on the moderating interaction effect on the mediating model. Combining insights from Preacher et al. (45) and Dong and Mao (46) on Bootstrap mediation analysis, we present the results of the moderated mediation analysis in **Table 4**. Firstly, psychache mediates the interaction effect of CT and AEE on depression (F = 12.453, p < 0.001, R2 = 0.212), and the interaction effect of CT and AEE on psychache is significant (β = 0.118, T = 2.853, p = 0.005); The effect of psychache on depression is moderated by PA (F = 70.244, p < 0.001, R2 = 0.793), and the interaction effect between psychache and PA on depression is significant (β = 0.090, T = -2.939, p = 0.004). Secondly, When divided into low and high groups based on the mean plus or minus one standard deviation of AEE and PA, it was found that the mediating effect of psychache was significant for university students with low AEE (*p* < 0.001), with confidence intervals of [0.051, 0.376], [0.047, 0.338], and [0.042, 0.306]; The mediating effect of psychache was also significant for university students with high AEE (*p* < 0.001), with confidence intervals of [0.275, 0.522], [0.254, 0.462], and [0.221, 0.422].

**Table 4:** Results of the Bootstrap mediated effects test with the PROCESS (*N*=476).

Model Summary: Multiple regression analysis of the mediator variable with the independent variable and the moderator mediator variable.	Psychache				
	R	R^2^	F	df	P
	0.461	0.212	12.453^***^	9.000	0.000
Variables	Effect	SE	T	LLCI	ULCI
CT	0.395^***^	0.045	8.701	0.306	0.484
AEE	0.133^**^	0.046	2.913	0.043	0.222
CT×AEE	0.118^**^	0.041	2.853	0.037	0.199
Model Summary: Multiple regression analysis of the mediator variable with the independent variable, the mediator variable, the moderator mediator variable, and the mediator moderator interaction variable.	Depression				
	R	R^2^	F	df	P
	0.793	0.629	70.244^***^	10.000	0.000
Variables	Effect	SE	T	LLCI	ULCI
CT	0.179^***^	0.034	5.284	0.113	0.246
Psychache	0.700^***^	0.033	21.002	0.635	0.766
PA	-0.057	0.032	-1.781	-0.120	0.006
Psychache×PA	-0.090^**^	-0.031	-2.939	-0.150	-0.030
Model Summary: Direct effect of the independent variable on the dependent variable after controlling for the mediator variable, the moderator mediator variable, and the mediator moderator interaction variable.	Depression				
Variables	Effect	SE	T	LLCI	ULCI
CT	0.179^***^	0.034	5.284	0.113	0.246
Mediator variable	AEE	PA	Effect	Boot LLCI	Boot ULCI
Psychache	Low	Low	0.218^***^	0.051	0.376
Psychache	Low	High	0.176^***^	0.042	0.306
Psychache	High	Low	0.402^***^	0.275	0.522
Psychache	High	High	0.325^***^	0.221	0.422

Model, 21; CT, childhood trauma; AEE, ambivalence over emotion expression; PA, physical activity. ^**^p < 0.01, ^***^p < 0.001.

Following Chen et al. (47) viewpoint, if none of the confidence intervals contains 0, then the indirect effect holds. This shows that in the chain of influence between CT and depression, the mediating effect of psychache, the moderating effect of AEE, and the moderating effect of PA are all significant. In **Table 3**, it reflects the explanatory power of independent variables on the dependent variable individually. The regression analysis in **Table 3** confirms that the total effect of CT on depression is 0.466. However, after controlling for the mediating variable of psychache and the moderating variables of AEE and PA, the direct effect of CT on depression decreases to 0.179. Therefore, it indicates that in the underlying mechanism of the relationship between CT and depression, the overall indirect effect of the three variables of AEE, psychache, and PA is 0.287, which accounts for 61.59% of the total effect, far greater than the direct effect.

**Figure 2:**
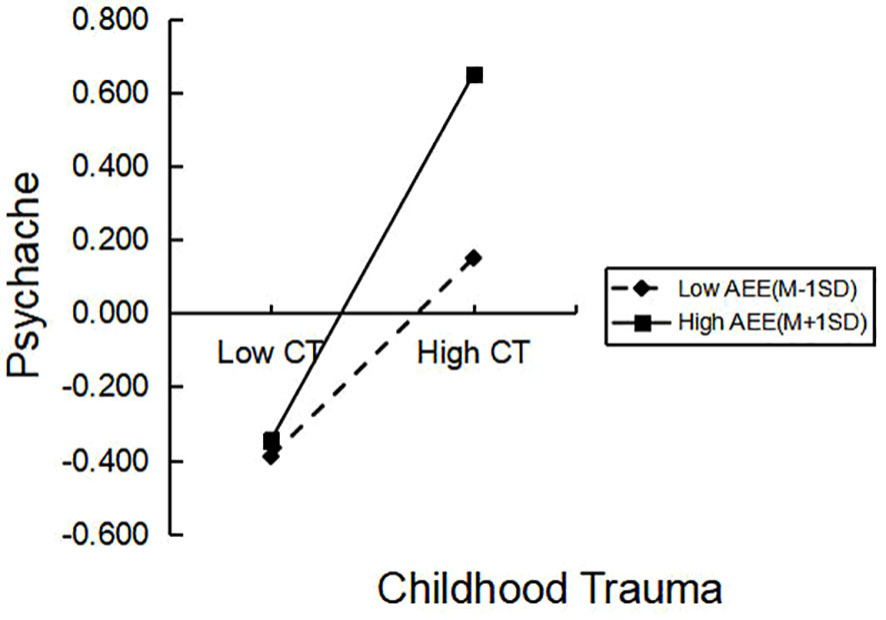
The moderating role of AEE in the relationship between CT and Psychache.

**Figure 3:**
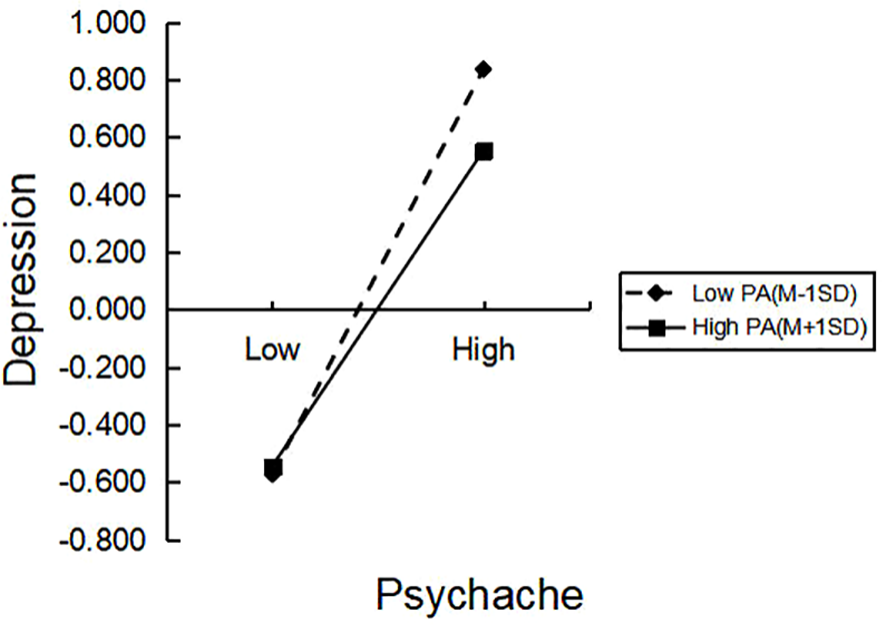
The moderating role of PA in the relationship between Psychache and Depression.

As shown in **Figure 2**, when AEE is low, CT has a significant predictive effect on psychache. When AEE is high, the association with CT on psychache is more significant, indicating that as the level of AEE increases, the predictive effect of CT on psychache is strengthened, thus supporting hypothesis 4a. In **Figure 3**, it can be seen that compared to the high level of PA, when the level of PA is low, psychache has a more significant predictive effect on depression. High-level PA weakens the impact of psychache on depression, thus supporting hypothesis 4b.

## Discussion

This study examined the relationship between CT and depression in university students, the mediating role of psychache between them, and the moderating role of AEE and PA. Firstly, the present study found a relationship between CT and depression among university students, consistent with other researchers and the present study’s hypothesis (10, 12). CT is a frequent early stressor factor that raises the risk of depression in early adulthood (48). The susceptibility-stress model of depression shows that susceptibility and stress interact to influence the onset of depression. Susceptibility is primarily concerned with personality, cognitive patterns, and coping styles. An individual’s external stressors involve both distant and recent stressors, such as traumatic childhood experiences and everyday life events. CT can influence susceptibility formation and have an impact on depression (49). The analysis showed that the effect of CT on depression among university students amounted to 0.466, with an explanatory power of 21.7%, a result that shows that university individuals who were abused in childhood are more prone to negative cognitive biases, which may be manifested in the form of denial of oneself, mistrust of others, and pessimism about the future, further exacerbating the depression.

Secondly, psychache is regarded as one of the important triggers of depression in the opelessness Theory of Depression. When individuals face setbacks and dilemmas, psychache is often accompanied by a pessimistic view of the future and doubts about their own abilities, producing pathways such as dysregulation of emotions and impaired social interactions, which have a direct or indirect impact on depressed mood and gradually lead to the emergence and exacerbation of depressive mood. Hypothesis 2 is further confirmed in this paper. Psychache was mediating the relationship between CT and depression, which also supported our hypothesis 3. The result is consistent with other researchers on the correlation between CT, psychache (14, 23), and depression (40 Experiencing a traumatic event in childhood can lead to an increase in an individual’s level of psychache. Individuals who experience CT develop an avoidance of self and harmful attributions of failure and environmental maladjustment, leading to self-avoidance and high aversion (17). Psychache ensues when the individual makes internal attributions and believes that the ego is responsible for the negative outcome (17). This painful feeling is difficult to relieve and can turn into depression.

The study found that AEE moderated the relational pathway between CT and psychache and that the effect of CT on psychache is more pronounced in university students with high AEE, which supports hypothesis 4a of our study. The findings are congruent with those of earlier studies that childhood neglect and abuse can lead to children’s AEE in adulthood (23). As a personality trait, individuals with high AEE traits are more inclined to adopt immature defense mechanisms (26). Individuals face problems inhibiting their potential for extreme stress management, generalizing and making pain severe, and leading to negative emotions and intense psychache (26). In particular, during the critical period of socialization, university students are constrained by the norms of “reasonableness” and “moderation” in their social interactions and cultural demands, and their emotional expressions may appear restrained and subtle (50). They will perceive their surroundings more negatively, thus deepening the individual’s distress (51). On the contrary, individuals with low AEE traits seldom experience significant psychache despite experiencing CT, as it seldom impedes their ability to manage high levels of stress effectively (30).

This study has shown that university students with high levels of PA have a weaker effect of psychache on depression compared to low levels of PA, which supports hypothesis 4b of our study. PA has shown significant improvement in individuals’ negative moods (52) and is a holistic means of coping regulation that can promote physical and mental health. The basic premise of PA for emotion regulation is that PA induces positive thoughts and emotions, which can counteract the effects of a negative state of mind (34). Individuals with low levels of PA are less able to control and regulate their emotions and have a poorer perception of negative emotions, which is more likely to lead to mental illness (53). Higher levels of PA are associated with fewer negative emotional experiences. Regular exercise contributes to releasing B-endorphins in the body to reduce pain and improve mood (54). In addition, physical activity acts as a form of distraction, self-catharsis, or vicarious relocation to divert undesirable emotions and reduce damaging emotional build up (55).

University students are highly vulnerable at this stage of their lives, and various external stressors can significantly impact their depression levels. They face high academic standards, recent adversities, and significant changes in their daily and social lives, which can place them in a chronic state of emotional stress. If perceived as insurmountable, these stressors can lead to stress, passivity, and depression (56). Personal characteristics also play a crucial role. Female students are more susceptible to emotional distress and exhibit higher rates of depression than male students, possibly due to differences in social roles and coping strategies. Only children often bear higher expectations and pressure from their families, leading to greater feelings of pressure and loneliness. Additionally, differences in academic programs lead to varying levels of stress and employment anxiety, with science students typically facing more significant challenges. Therefore, further research is needed to better understand the mechanisms affecting depression among university students.

Our study has great significance. Theoretically, this study is from the view of AEE and PA. It provides evidence in support of the integrated susceptibility-stress model of depression. A moderated mediation model was constructed to explore the relationship between CT, psychache, AEE, PA, and depression. To a certain extent, it explains the underlying mechanisms by which CT associated with depression in university students. In practice, the protective effect of mood regulation on depression provides a new perspective on the prevention and intervention of depression. Some strategies can be developed in the future based on these influencing factors. First, we can avoid the source of trauma and intervene with individuals who have experienced CT. Second, we can encourage university students to be aware of their raw emotions and to interact socially by describing their feelings to significant others at the right time. Screening for people with high levels of AEE for necessary attention and psychological support after experiencing CT, such as such as mindfulness therapy (57) and cognitive behavioral intervention therapy (58). Third, to promote active student participation in PA, utilizing it as a means of distraction and self-catharsis to alleviate the accumulation of negative affect.

### Limitations

Some limitations should also be considered in this study. Firstly, this study is cross-sectional, and although it has been tested in reverse and does not hold, it still does not confirm the causal relationships between the core variables hypothesized. Future research should adopt a more precise experimental design or a longitudinal follow-up study design to investigate further the relationship and mechanisms between CT and depression. Secondly, this study used self-report methods to assess CT, psychache, and depression with some recall bias, and future investigations should adopt a multimodal approach and incorporate objective measures to enhance the validity of the data obtained. With regards to population selection, future studies could expand their scope by exploring other groups, such as the non-college-bound population.

## Conclusions

This study investigated the relationship between CT, AEE, psychache, PA, and depression in university students and found a significant positive relationship between CT and depression. CT can also influence depression through a partially mediated role of psychache, with AEE moderating the relationship between CT and depression. The higher the AEE, the stronger the effect of CT on psychache, and the weaker the opposite. PA moderated the positive relationship between psychache and depression, with higher levels of PA having a weaker level of depression and vice versa. The study’s findings have important implications for enhancing university students’ mental health and reducing depression.

## Data availability statement

The original contributions presented in the study are included in the article/**Supplementary Material**. Further inquiries can be directed to the corresponding author.

## Ethics statement

The studies involving human participants were reviewed and approved by the School of Public Health Ethics Committee at Cheeloo College of Medicine of Shandong University (20190912). The participants provided their written informed consent to participate in this study.

## Author contributions

SC: Conceptualization, Funding acquisition, Data curation, Formal analysis, Investigation, Methodology, Project administration, Software, Supervision, Validation, Visualization, Writing – original draft, Writing – review & editing. TF: Conceptualization, Data curation, Investigation, Methodology, Project administration, Software, Supervision, Visualization, Writing – original draft, Writing – review & editing. YW: Data curation, Formal analysis, Methodology, Project administration, Supervision, Validation, Visualization, Writing – original draft, Writing – review & editing. GS: Conceptualization, Data curation, Formal analysis, Investigation, Methodology, Project administration, Resources, Software, Supervision, Validation, Visualization, Writing – original draft, Writing – review & editing.
